# Application of graph frequency attention convolutional neural networks in depression treatment response

**DOI:** 10.3389/fpsyt.2023.1244208

**Published:** 2023-11-17

**Authors:** Zihe Lu, Jialin Wang, Fengqin Wang, Zhoumin Wu

**Affiliations:** College of Physics and Electronics Science, Hubei Normal University, Huangshi, China

**Keywords:** classification, depression treatment response, EEG, graph convolutional neural networks, frequency attention

## Abstract

Depression, a prevalent global mental health disorder, necessitates precise treatment response prediction for the improvement of personalized care and patient prognosis. The Graph Convolutional Neural Networks (GCNs) have emerged as a promising technique for handling intricate signals and classification tasks owing to their end-to-end neural architecture and nonlinear processing capabilities. In this context, this article proposes a model named the Graph Frequency Attention Convolutional Neural Network (GFACNN). Primarily, the model transforms the EEG signals into graphs to depict the connections between electrodes and brain regions, while integrating a frequency attention module to accentuate brain rhythm information. The proposed approach delves into the application of graph neural networks in the classification of EEG data, aiming to evaluate the response to antidepressant treatment and discern between treatment-resistant and treatment-responsive cases. Experimental results obtained from an EEG dataset at Peking University People's Hospital demonstrate the notable performance of GFACNN in distinguishing treatment responses among depression patients, surpassing deep learning methodologies including CapsuleNet and GoogLeNet. This highlights the efficacy of graph neural networks in leveraging the connections within EEG signal data. Overall, GFACNN exhibits potential for the classification of depression EEG signals, thereby potentially aiding clinical diagnosis and treatment.

## 1 Introduction

Depression, a widespread mental disorder, is predominantly assessed for treatment response using subjective clinical data. To overcome this constraint, the scientific community has begun to investigate the potential of biomedical signals as supplementary diagnostic instruments. Comparing to fMRI (1, 2), Electroencephalography (EEG) signals, which are non-invasive, safe, and offer high-resolution, can depict the dynamic alterations in brain neural activity. Nevertheless, the analysis of EEG data faces multiple obstacles, such as the presence of noise and interference, individual variability, and non-stationary and nonlinear signals, among others (3). To tackle these challenges, researchers are exploring innovative methods and techniques to enhance the precision and efficiency of EEG signal processing, ultimately delivering more objective and accurate guidance for diagnosing and treating depression (3).

Graph convolutional neural networks (GCN) have garnered significant interest in recent years as a powerful deep learning model for processing complex signals and classification tasks across various fields. However, the utilization of GCNs for classifying EEG signals remains limited. The most salient related work are as following, Song et al. (4) and Jang et al. (5) have shown the successful application of GCNs in EEG emotion recognition and video identification, respectively, while Li et al. (6) introduced an information aggregation method to transmit information between graph convolutional layers. Zhao et al. (7) proposed a linear graph convolutional network for seizure identification, achieving an accuracy of 99.30% with the use of focal loss to address data imbalance. Recently, to improve depression detection, Zhu et al. (8) proposed a Graph Input layer attention Convolutional Network (GICN), which incorporated a learnable weight matrix in the input layer of the GCN, achieving a 96.50% accuracy for recognition of depression and normal with 10-fold cross-validation. Unlike recurrent neural networks (RNNs), which are well-suited for sequential data, GCNs demonstrate superior performance in processing nonlinear signals by capturing their inherent nonlinear relationships. Moreover, GCNs offer advantages such as the ability to effectively exploit node relationships, accommodate variable-sized inputs, process multiple data types simultaneously, and exhibit end-to-end characteristics. These attributes contribute to enhanced classification performance, enable the handling of unstructured data, provide greater flexibility, and reduce information loss and error introduced during manual feature extraction (9).

While traditional graph attention methods have demonstrated remarkable outcomes in several applications by automatically learning attention on data, they are limited in terms of network connections, which are mostly confined to the time-domain space, lacking connections based on the frequency domain. Nonetheless, this approach lacks specificity and controllability, as it typically computes attention weights based on intrinsic features of data, without explicit guidance for particular tasks, possibly highlighting irrelevant features and reducing the model's performance. Furthermore, traditional methods require extensive trial-and-error and parameter adjustments to achieve better attention, further limiting the model's controllability. Therefore, despite the ability of traditional graph attention methods to learn attention on data automatically, they lack specificity and controllability for particular tasks. To overcome this limitation, we propose the Graph Frequency Attention Convolutional Neural Network (GFACNN), which utilizes signal frequency information as attention weighting information to guide the model towards EEG rhythm information. This new deep learning model is inspired by the channel frequency attention mechanism (10) and aims to address this limitation.

## 2 Methodology

**Figure 1:**
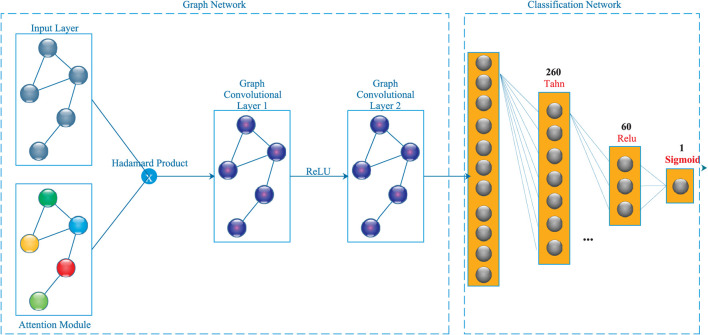
The architecture of GFACNN.

This section introduces the GFACNN model. Figure 1 illustrates the architecture of GFACNN.

### 2.1 Attention module

The parameter vector $\vec{a}$ in the GFACNN is a nonlearnable attention mechanism for each node. It is a vector of weights to determine the importance of each feature dimension in the computation of attention coefficients.

First, the frequency of channel **x**_*i*_ is obtained by the discrete-time Fourier transform (DTFT) algorithm:


(1) $$\begin{array}{l} X\left(\omega \right)=\overset{\infty }{\underset{n=-\infty }{\sum }}x_{n}{\text{e}}^{-\text{i}\omega n}, \end{array}$$

where ${\left\{x_{n}\right\}}_{n=-\infty }^{\infty }$ is the *i*th channel **x**_*i*_.

The power *E* of the spectrum is calculated as:


(2) $$\begin{array}{c} F\left({\text{e}}^{j\omega }\right)=a+ib,\\ \left|F\left({\text{e}}^{j\omega }\right)\right|=\sqrt{a^{2}+b^{2}},\\ E=\frac{1}{2\pi }\int_{-\pi }^{\pi }{\left|F\left({\text{e}}^{j\omega }\right)\right|}^{2}d\omega . \end{array}$$

The mean power Ē for the channel **x**_*i*_ is then calculated and indexed as *i*. And the average power values of all channels are constructed into one attention vector according to their indexes.


(3) $$\vec{a}=[{\bar{E}}_{0},{\bar{E}}_{1},\cdots ,{\bar{E}}_{n}]$$

Finally, the attention vector $\vec{a}$ is normalized to [0.1, 1] by max-min normalization. It is applied to the input node features as the weight in terms of the Hadamard product.

### 2.2 Graph frequency attention convolutional neural network

GFACNN is a novel graph convolutional neural network specifically designed for classification and object detection. The network's architecture comprises of an attention-based input layer that processes the input data and extracts frequency relevant feature representations (Section 2.1). This input layer employs an attention mechanism to identify and learn the significance of different regions in the EEG data, thereby enhancing the feature extraction process's efficacy. It represent the original graph data as a node feature matrix X, where *X*_*i*, :_ represents the feature vector of the i-th node.

The network also features two graph convolutional layers that leverage graph convolution operations to extract the features from the input data. These layers contain multiple graph convolution units that update the node's feature representation by aggregating information from the neighboring nodes:


(4) $$H^{\left(l\right)}=\sigma \left({\tilde{D}}^{-\frac{1}{2}}\tilde{A}{\tilde{D}}^{-\frac{1}{2}}H^{\left(l-1\right)}W^{\left(l-1\right)}\right)$$

where Ã = *A*+*I* is the adjacency matrix A with self-loops added, $\tilde{D}$ is the diagonal matrix where each diagonal element ${\tilde{D}}_{i,i}=\underset{j}{\sum }{Ã}_{i,j}$, σ is the activation function, and *W*^(1)^ is the weight matrix at layer *l*.

Moreover, GFACNN consists of three fully connected layers, which have a unique hourglass-shaped structure (11). These layers' primary function is to map the features extracted by the graph convolutional layers to the respective target categories.

Despite its relatively intricate structure, GFACNN offers the advantage of end-to-end learning and feature extraction from input data, facilitated by the graph convolutional neural networks. This allows it to significantly enhance the effectiveness of depression treatment response classification tasks.

### 2.3 Training of GFACNN

The training procedure involves the computation of the Hadamard product between the input data and the feature representations pertinent to frequency, as expounded in Section 2.1. Subsequently, this computed product traverses through two layers of the graph convolutional neural network. At each of these layers, a graph convolution operation is implemented. This operation encompasses the aggregation of information emanating from neighboring nodes, subsequently influencing the update of node features. Following each instance of graph convolution operation, an activation function (illustrated in Figure 1) is applied in an element-wise manner to the amalgamated information. The anticipated outcome is generated through the final quartet of fully connected layers, as depicted in Figure 1, each comprising a configuration of neurons. The discrepancy between the foreseen output and the factual target labels is gauged through the medium of a designated loss function. To optimize this process, the Stochastic Gradient Descent optimizer is brought into play. This optimizer's objective is the minimization of the Mean Squared Error loss function with a learning rate of 0.01, consequently contributing to the enhancement of the model's performance. This optimization endeavor is facilitated through the continual refinement of the model's parameters, a feat achieved via the Backpropagation algorithm. This iterative process of training persists until a specified termination criterion is satisfied (notably, at Epoch 100, with a batch size of 30, and early termination employing a Patience of 10). Upon the culmination of the training phase, an evaluation of the model's efficacy is conducted through its deployment on an autonomous testing dataset. This assessment offers an impartial estimation of the model's capacity to generalize to hitherto unseen data instances.

## 3 Results

The experiments in this section serve as a validation and assessment of the classification of the proposed model. We first introduce the dataset (see Section 3.1) after describing experimental platform utilized in the experiments. Next, rhythmic information in EEG of depression treatment response is visualized (see Section 3.2). Finally, the classification of GFACNN is evaluated using accuracy, sensitivity and specificity (see Section 3.3). The experiments were conducted on a desktop with an Intel i7 CPU at 3.33 GHz, an Nvidia RTX 2080Ti GPU, 64GB RAM, and Windows 7. This system enabled consistent testing conditions.

### 3.1 Dataset

The EEG dataset was obtained from 17 patients with major depressive disorder (MDD) at Peking University People's Hospital. The dataset was recorded simultaneously through 20 channels (Fp1, Fp2, F3, F4, F7, T3, T5, C3, C4, Fz, Cz, Pz, F8, T4, T6, P3, P4, O1, O2, ECG) at a sampling rate of 256 Hz using 19 electrodes and one electrocardiograph. MDD subjects receiving antidepressant treatment were selected from the hospital. In this study, all 17 patients received escitalopram oxalate tablets (10 mg), with 7 subjects being classified as non-responsive. The time window was set to 1024 samples (4 s), resulting in a total sample space of 20,851 segments (12482 treatment-responsive and 8369 treatment-resistant).

The adjacency matrix *A*∈*R*^*n* × *n*^ of the GFACNN is constructed using the topological structure of brain electrical channels. In this matrix, *n* represents the number of channels in the brain electrical signal and each element value *A*_*ij*_ represents the connection weight between channels *i* and *j*. The spatial relationship between channels and brain regions is taken into account in the adjacency matrix *A*. The Pearson correlation coefficient is used to obtain the correlation coefficient *R*_*ij*_ between two channels *i* and *j*, while the Heterogeneous Matrix Similarity Measurement (HMSM) method (12) is employed to determine the similarity *H*_*ij*_ between pairs of brain regions. The value of *A*_*ij*_ is then calculated as *A*_*ij*_ = *R*_*ij*_+λ*H*_*ij*_, where λ represents a calibration constant greater than 0 (typically 0.5). The attention for each channel will subsequently be applied to the adjacency matrix.

### 3.2 Frequency analysis of treatment response

**Figure 2:**
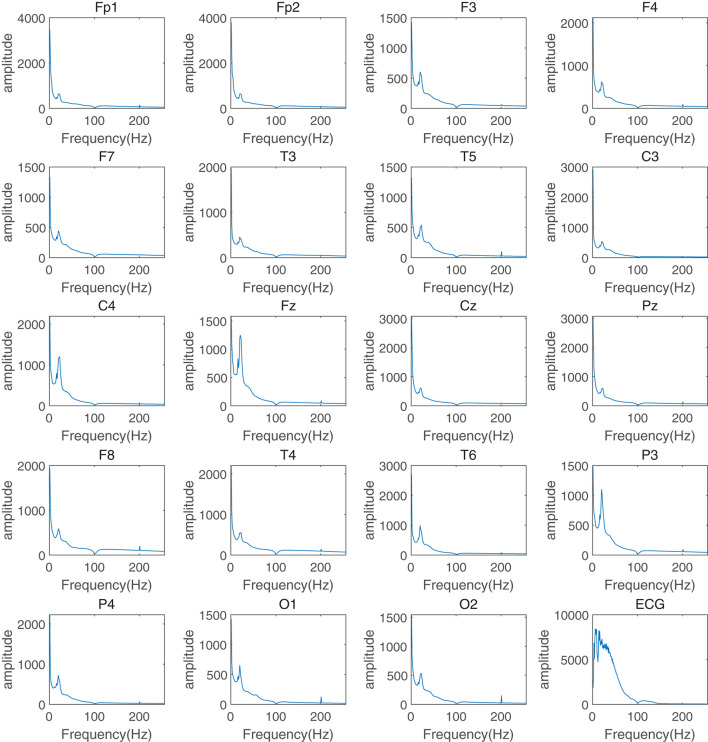
Time-frequency analysis of different channels in the treatment-responsive group for depression treatment response.

**Figure 3:**
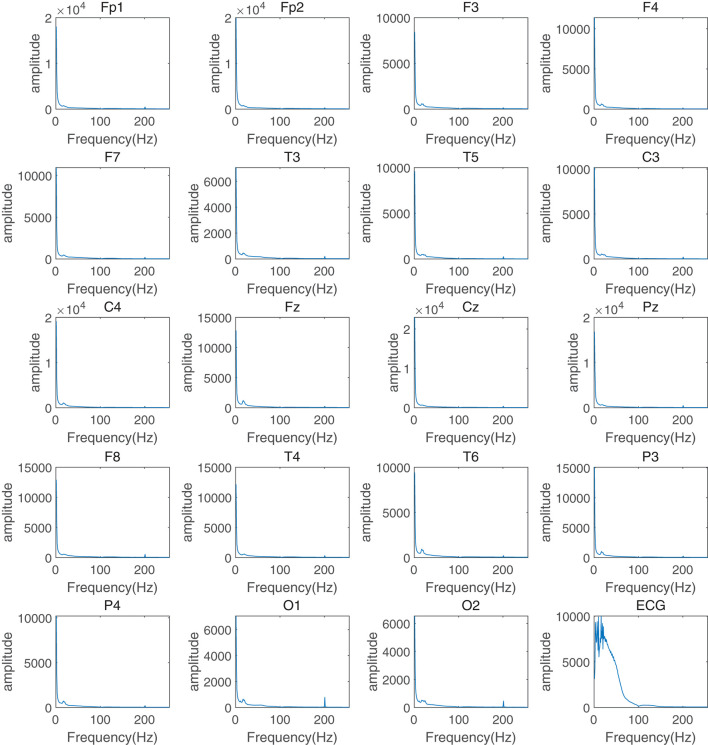
Time-frequency analysis of different channels in the treatment-resistant group for depression treatment response.

This section of the experiment aimed to evaluate the important role of frequency information in assessing the response to depression treatment and to explain why this study used frequency information as the attention mechanism in a graph neural network. First, the EEG data were preprocessed to remove artifacts, pulse noise, and baseline drift. Continuous EEG data were then segmented into multiple 4-second data segments for fast FFT with time-frequency analysis. FFT transforms were calculated to obtain the average power for pre-treatment and post-treatment groups. The average frequency components and corresponding power distribution in the δ (0.5–4Hz), θ (4-8Hz), α (8-13Hz), and β (13–30Hz) frequency bands (Figure 2 for the treatment-responsive group, Figure 3 for the treatment-resistant group) were generated for different channels across groups to evaluate depression treatment response.

A comparison of spectral power graphs (Figures 2, 3) reveals that the power (amplitude illustrated in figures) of the treatment-resistant group is significantly higher than that of the treatment-responsive group. This observation may be attributed to several factors: (1) Abnormal brain electrical activity: Patients with depression often exhibit abnormal brain electrical activity, characterized by either heightened or diminished excitability relative to healthy individuals. This can result in increased power within certain frequency bands. In treatment-resistant patients, these abnormalities may be more severe, leading to further increases in spectral power (13). (2) Spectral line amplification: Increased amplitudes of select frequency components within certain bands can result in an overall increase in energy. This phenomenon may be associated with enhanced synchronization or oscillation activity of brain waves (14). (3) Effects of therapeutic drugs: Antidepressants can modulate abnormal brain electrical activity by targeting neurotransmitter systems (15, 16). However, in treatment-resistant patients, these drugs may be treatment-resistant or produce unexpected side effects, leading to further changes in the brain electrical spectrum.

Another phenomenon in the figures is that all channels in the treatment-responsive group show an “impulse response” in the α frequency band especially for channels F3, C4, Fz, P3, and P4, while it is not found in the treatment-resistant group. The reasons for this may be as follows: First, α waves are related to attention and alertness (17). Treatment-responsive can improve the patient's attention and alertness, causing a temporary drop in α wave power after the stimulus appears, that is, an “impulse response”. Patients with treatment-resistant have difficulty improving their attention, so this phenomenon does not occur. Second, α waves are related to emotional regulation (18). Antidepressant treatment can improve emotional regulation. When an external stimulus occurs, α waves will temporarily decrease to adjust the emotional state and produce an “impulse response”. Patients with treatment-resistant treatment have more severe emotional regulation disorders. The regulatory mechanism of α waves is impaired and it is not easy to produce this phenomenon. Finally, α waves are related to functional connections between brain regions (19). Treatment-responsive can enhance functional connections and synchrony between brain regions. When external stimuli occur, α waves between brain regions will temporarily lose coordination and then quickly recover, producing a significant “impulse response”. For the treatment-resistant group, it is difficult to repair the connection between brain regions and it is not easy to produce this phenomenon.

### 3.3 Performance of depression treatment response

**Figure 4:**
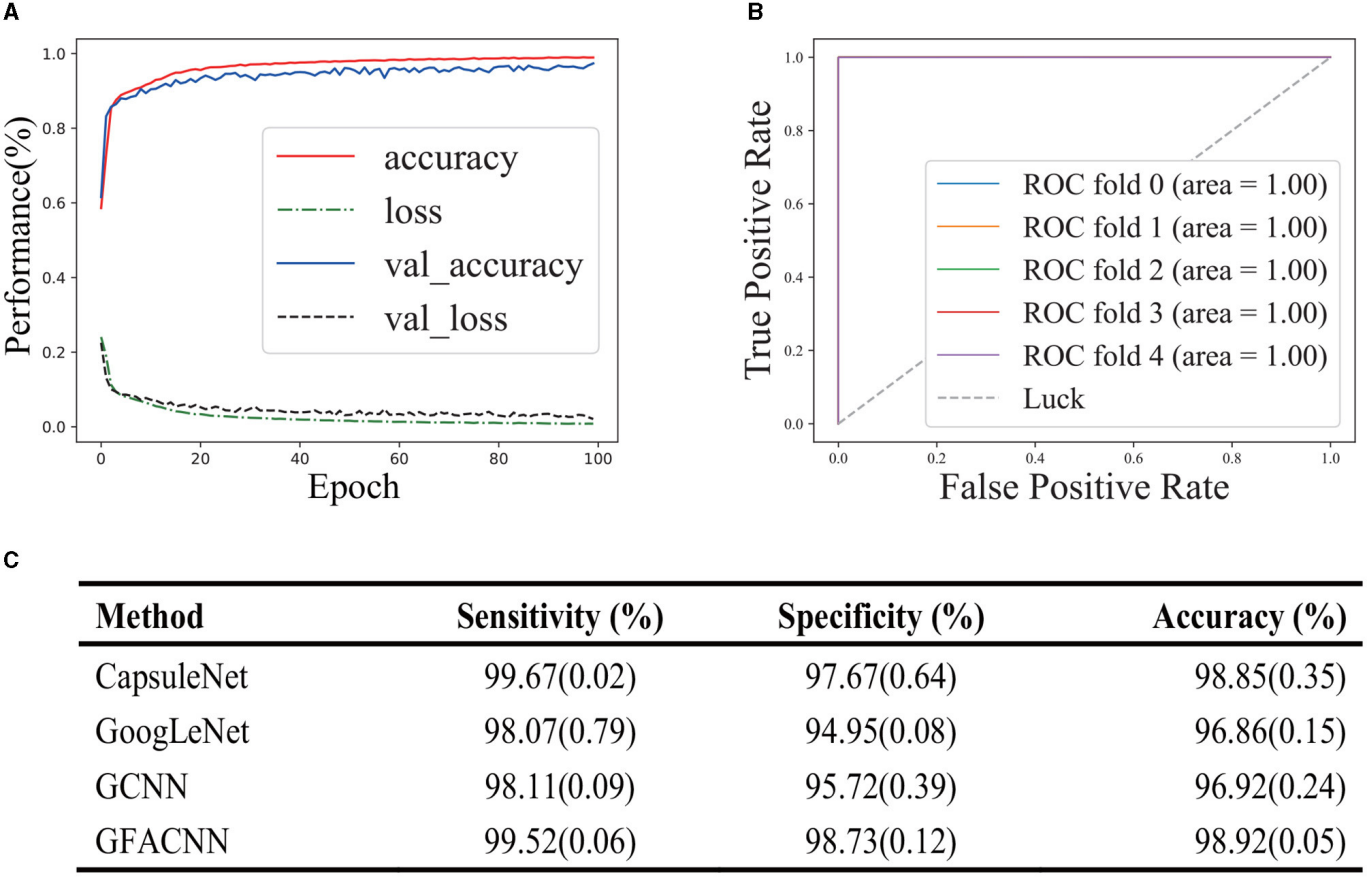
Performance evaluation of the model in the efficacy of antidepressant treatment. The number in parentheses represents the standard deviation. **(A)** learning curve provide insight into the dependence of a classifier's generalization performance on the training set and validation set. **(B)** ROC (Receiver Operating Characteristic) curve illustrated the performance of a classification model at all classification thresholds. **(C)** Performance comparison on Depression Treatment Response.

In this section, we assessed the ability of GFACNN to discriminate between treatment responses in patients receiving antidepressant therapy at Peking University People's Hospital. We employed a 5-fold cross-validation approach to train our classifier and evaluated its performance on an independent test set. To monitor the training process, we utilized learning curves and assessed the model's predictive capabilities using receiver operating characteristic (ROC) curves and the area under the curve (AUC). Our results, depicted in Figures 4A, B, demonstrate that our classifier exhibited stable learning without overfitting or underfitting. This suggests that our approach is generalizable. Furthermore, our classifier performed exceptionally well on the test set, indicating its high discriminative power for treatment responses. Taken together, our findings suggest that GFACNN may serve as a valuable tool for evaluating the efficacy of antidepressant therapy.

Finally, the model's classification performance was assessed on the hold-out test set. Our approach attained 98.92% accuracy, 99.52% sensitivity, and 98.73% specificity, as summarized in Figure 4C. In comparison to the foundational classifiers, which encompass GCNN (the GFACNN variant without the frequency-channel attention module), capsule networks, and GoogleNet, our proposed methodology has evidenced a marked advancement in terms of performance. The attention mechanism module in GFACNN leads to an approximate 2% enhancement in performance. Furthermore, the GFACNN displays a reduced standard deviation, suggesting a heightened concentration of performance values around its mean and a decreased level of dispersion.

## 4 Discussions and conclusions

In this section, we offer an extensive examination of EEG channels utilized in the construction of our GFACNN. These EEG channels bear multifaceted implications across biological, psychological, and psychiatric domains, enriching our understanding of depression treatment response.

**EEG channels:** One of the pivotal aspects of this study is the incorporation of channels that closely align with those reported in existing literature. This alignment provides a robust foundation for the exploration of neural mechanisms underlying the response to depression treatment. Our findings are consistent with the work of Ressler (20), who highlighted the pivotal role of these channels in the modulation of neural activity associated with mood regulation. Moreover, the neurophysiological patterns observed in these channels in the rostral anterior cingulate cortex (ACC) resonate with the antidepressant response (21), corroborating the relevance of our channels to depression-related neural processes.

Incorporating the EEG channels F3, C4, Fz, P3, and P4, which primarily correspond to the prefrontal and frontal regions (22), into the GFACNN architecture has yielded compelling evidence of alignment with well-established neural pathways associated with depressive conditions. Noteworthy research indicates the involvement of prefrontal connectivity (23) and frontal EEG connectivity (21) in emotional regulation and mood disorders at the level of brain regions, thereby underscoring its relevance in elucidating treatment outcomes for depression. Furthermore, at a finer granularity of channel-level analysis, the investigation revealed hypoactivity within the left frontal hemisphere for F3 and F4, accompanied by a global elevation in alpha power in depressive disorders, as highlighted by Horato et al. (18). Furthermore, in the context of EEG signals from F3, P3, and P4, a robust correlation emerged between Phase-amplitude coupling delta-beta (dPAC-DB) and Mood Disorder Questionnaire (MDQ) scores, both pre- and post-treatment, as detailed by Kesebir et al. (24).

The incorporation of these meaningful channels not only adds to the robustness of our model but also enhances the interpretability of our results. The utility of these channels in classification is grounded in their ability to capture neural activity patterns that are inherently linked to depression and its treatment.

**Clinical implications of classification:** The implications of our findings extend beyond the realm of research to the clinical domain. By identifying and highlighting the neural patterns most indicative of depression treatment response, our GFACNNs offer clinicians an objective tool to aid in treatment planning and decision-making. The accuracy and interpretability of our model's predictions pave the way for personalized treatment strategies, ensuring that interventions are tailored to an individual's neural response profile.

**Limitations:** While our study presents promising insights, we acknowledge the presence of several limitations that warrant thorough consideration. Notably, one of these limitations pertains to the sample size within our dataset, which, despite meticulous curation, could potentially exert influence on the generalizability of our findings. Furthermore, the absence of longitudinally tracked follow-up data constrains our capacity to evaluate the enduring effects of the administered treatment regimen. It is also important to recognize that the utilization of self-reported metrics for certain clinical parameters introduces the prospect of recall bias. The conscientious addressal of these limitations in forthcoming investigations will undoubtedly enrich the holistic comprehension of the intricate neural mechanisms that underlie the response to depression treatment.

To summarize, the findings from our research suggest that GFACNN possesses substantial promise in assessing the effectiveness of antidepressant treatment. The potential clinical applications of our model are extensive, paving the way for more precise and efficacious interventions. Despite recognizing the constraints of our study, we maintain a positive outlook that our efforts provide a robust groundwork for future progress in this crucial research domain.

## Data availability statement

The original contributions presented in the study are included in the article/supplementary material, further inquiries can be directed to the corresponding author.

## Ethics statement

Ethical approval was not required for the studies involving humans because the original data collection followed proper ethical protocols and approvals. The data is collected from hospitals and does not violate local laws. The studies were conducted in accordance with the local legislation and institutional requirements. Written informed consent for participation was not required from the participants or the participants' legal guardians/next of kin in accordance with the national legislation and institutional requirements because the original data collection followed proper ethical protocols and approvals.

## Author contributions

FW contributed to the conception of the study, the reagents, materials, and analysis tools, conceived and designed the experiments, and analyzed the data. ZL, JW, and ZW performed the experiments. All authors contributed to the article and approved the submitted version.
